# FleQ finetunes the expression of a subset of BrlR-activated genes to enable antibiotic tolerance by *Pseudomonas aeruginosa* biofilms

**DOI:** 10.1128/jb.00503-24

**Published:** 2025-04-30

**Authors:** Victoria I. Oladosu, Karin Sauer

**Affiliations:** 1 Department of Biological Sciences, Binghamton University 14787, Binghamton, New York, USA; 2 Binghamton Biofilm Research Center, Binghamton University 14787, Binghamton, New York, USA; Geisel School of Medicine at Dartmouth, Hanover, New Hampshire, USA

**Keywords:** BrlR, DNA binding, immunoblot, pulldown, biofilm-MBC, resistant to killing, BACTH, protein-protein interaction

## Abstract

The transcriptional regulator FleQ contributes to *Pseudomonas aeruginosa* biofilm formation by activating the expression and biosynthesis of matrix exopolysaccharides in a manner dependent on c-di-GMP. However, little is known about the role of FleQ in the antibiotic tolerance phenotype of *P. aeruginosa* biofilms. Inactivation of *fleQ* impaired biofilm formation and rendered biofilms susceptible to tobramycin and norfloxacin. The phenotypes were similar to biofilms inactivated in *sagS* encoding the orphan sensor SagS that promotes the switch from planktonic to biofilm growth via BfiSR and antibiotic tolerance via BrlR. While FleQ was found to contribute to biofilm formation independently of SagS and BfiSR, FleQ instead converged with SagS-dependent regulation at the level of BrlR. This was supported by multicopy expression of *sagS* failing to restore biofilm antibiotic tolerance by *ΔfleQ* to wild-type levels (and *vice versa*) and by biofilms formed by the *ΔfleQΔsagS* double mutant being as susceptible as *ΔfleQ* and *ΔsagS* biofilms. Increased antibiotic susceptibility was independent of BrlR abundance or BrlR DNA binding but coincided with significantly reduced transcript abundance of the BrlR-activated *mexCD-oprJ* and PA1874-77, encoding an ABC transporter previously shown to contribute to the tolerance of biofilms to tobramycin and norfloxacin. FleQ- dependent regulation of gene expression was indirect. Co-immunoprecipitation and BACTH assays indicated FleQ to interact with SagS via its HisKA-Rec domain, likely suggesting FleQ and SagS to likely work in concert to enable biofilm antibiotic tolerance**,** by finetuning the expression of BrlR activated genes.

**IMPORTANCE:**

In *P. aeruginosa*, FleQ inversely regulates the expression of genes encoding flagella and biofilm matrix components, including exopolysaccharide (Pel, Psl) in a manner dependent on the levels of c-di-GMP. Our findings expand on the role of FleQ from regulating the transition to the biofilm mode of growth to FleQ contributing to the antimicrobial tolerance phenotype of biofilms, by FleQ affecting the expression of PA1874-77, a downstream target of the SagS-dependent transcriptional regulator BrlR. Importantly, our findings suggest FleQ works in concert with SagS, likely via FleQ-SagS protein-protein interactions, to enable the formation of inherently tolerant *P. aeruginosa* biofilms.

## INTRODUCTION

Biofilms are characterized by bacteria growing in surface-associated communities or non-adherent aggregates of cells enclosed in a self-produced exopolysaccharide matrix (1). These sessile communities are characterized by heightened tolerance to antimicrobial agents and host immune response, with biofilms having been reported to be up to 1,000 times less susceptible to antimicrobial compounds when compared to their planktonic counterparts (2), and are the root cause for many chronic infections (3, 4). For example, biofilm formation by the opportunistic pathogen *Pseudomonas aeruginosa* is responsible for a substantial proportion of human nosocomial infections such as community-acquired pneumonia, burn wounds, chronic catheter-associated infections, foot ulcers, and corneal and mechanical ventilation-related infections (5–8), as well as chronic biofilm-associated infections within the lungs of people with cystic fibrosis (9).

In *P. aeruginosa*, the transition from the planktonic to the biofilm mode of growth coincides with the inverse regulation of genes encoding flagella and biofilm matrix components, including exopolysaccharide (Pel, Psl, alginate) and adhesins (e.g., CdrA) (10–14), as well as induction of antibiotic tolerance mechanisms (including efflux-pumps even when biofilms were grown in the absence of antibiotics) and increased levels of virulence determinants (15–17). The phenotypic changes noted upon transition to the sessile mode of growth are driven by multiple signaling systems such as the Rhl, Las, and PQS that are essential for the regulation of virulence and multi-cellular behaviors in response to quorum-sensing signaling molecules (18–20). Additionally, the transition to the multi-cellular mode of growth is regulated by the coordinated work of regulatory proteins interacting within large signaling networks such as LadS/RetS/GacAS/Rsm (Gac/Rsm), HptB, Wsp, and Pil-Chp systems that fine-tune alterations in the intracellular level of cyclic di-GMP (c-di-GMP) and, to a certain extent, levels of cyclic AMP (21–26). C-di-GMP is a ubiquitous bacterial second messenger that has emerged as a key regulator of biofilm formation, capable of regulating a myriad of cellular functions in bacteria including group behavior such as motility, surface attachment, and virulence (27–29). Additional regulators enabling the transition from the planktonic to the biofilm mode of growth include the two component systems SagS (25, 30, 31) and the transcriptional regulator FleQ (13, 14, 32). The sensor-regulator hybrid SagS has been linked to Gac/Rsm signaling and c-di-GMP regulation, with SagS facilitating biofilm development and the activation of biofilm-associated antimicrobial tolerance via two distinct pathways (reviewed in reference 25). Specifically, SagS promotes the switch from planktonic to biofilm growth via phospho-signaling to the BfiSR two-component regulatory system (TCS), which, in turn, contributes to the regulation of small regulatory RNA (sRNA) levels (30, 31). In addition, via a separate mechanism, SagS regulates the switch from an antimicrobial susceptible to a highly tolerant state via regulation of c-di-GMP levels and subsequent activation of BrlR, a c-di-GMP responsive transcriptional regulator of biofilm resistance (33–36). Specifically, SagS indirectly regulates *brlR* expression and BrlR function via the diguanylate cyclase PA3177 (35). The activation of PA3177 leads to the production of c-di-GMP, which subsequently binds to and activates BrlR (33–35).

The transcription factor FleQ contributes to biofilm formation by inversely regulating genes encoding flagella and exopolysaccharides in a manner dependent on the secondary messenger cyclic di-GMP (c-di-GMP) (14). Specifically, FleQ activates the expression of genes involved in biofilm formation when intracellular levels of c-di-GMP are high, while ceasing the expression of genes associated with the motile lifestyle, including flagellar gene expression (reviewed in 37). While the role of FleQ in the transition from planktonic to the biofilm mode of growth has been well established, little is known about the role of *P. aeruginosa* FleQ in the formation of antibiotic tolerant biofilms. A recent study indicated the biofilm matrix to reduce the penetration of positively charged antibiotics such as tobramycin, but not neutral antibiotics (38). While the matrix component responsible for sequestering tobramycin was not elucidated, tobramycin penetration was increased by the addition of cations in a dose-dependent manner, likely suggesting an involvement of charged matrix components such as Pel or eDNA, but not Psl. FleQ contributes to the biosynthesis of Pel and Psl matrix polysaccharides (13). However, it is not known whether FleQ contributes to the antibiotic susceptibility phenotype of biofilms. The goal of this study was to determine the role of FleQ in the formation of drug tolerant biofilms and determine whether FleQ intersects with other regulatory systems to enable the transition from the planktonic to the biofilm mode of growth and subsequent biofilm formation.

## RESULTS

### FleQ contributes to the *P. aeruginosa* biofilm architecture

FleQ is known to inversely regulate the expression of genes encoding flagella and exopolysaccharides in a manner dependent on the secondary messenger cyclic di-GMP (c-di-GMP) (13, 14, 32) and, thus, modulating motility and biofilm formation by *Pseudomonas aeruginosa*. To further explore the role of FleQ in the formation of antibiotic tolerant biofilms, we first assessed the ability of a *P. aeruginosa* mutant strain inactivated in *fleQ* to form biofilms under flowing conditions.

**Fig 1:**
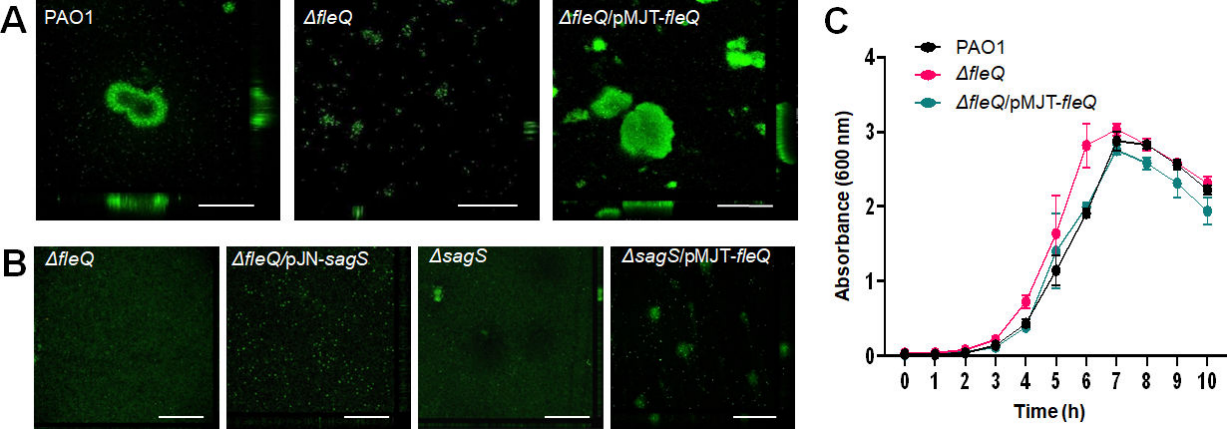
FleQ and SagS are both required for biofilm formation. Biofilms were grown in flow cells under flowing conditions for 6 days. Biofilms were stained using *Bac*Light LIVE/DEAD prior to visualizing the biofilm architecture by confocal microscopy. (**A**) Representative confocal images of biofilms by *P. aeruginosa* PAO1, *ΔfleQ* and *ΔfleQ*/pMJT-*fleQ*. (**B**) Representative confocal images of *ΔfleQ* and *ΔfleQ* expressing sagS (*ΔfleQ*/pJN-*sagS*) and *ΔsagS* expressing *fleQ* (*ΔsagS*/pMJT-*fleQ*). White bar = 100 µm. All experiments were performed in triplicate. (**C**) Growth curves. *P. aeruginosa* PAO1, *ΔfleQ,* and *ΔfleQ*/pMJT-*fleQ* were grown in VBMM at 37°C and 220 rpm and the absorbance was determined at 600 nm. Experiments were carried out in triplicate. Error bars indicate standard deviations.

Biofilm formation was evaluated post 6 days of growth under continuous flow conditions. Visual analysis of the biofilm structure indicated ∆*fleQ* to form unstructured, flat surface-associated bacterial communities, while biofilms formed by wild-type PAO1 featured large microcolonies (Fig. 1A). COMSTAT analysis confirmed the stark difference in biofilm biomass accumulation and biofilm height (Table 1). Multicopy expression of *fleQ* restored biofilm formation by ∆*fleQ* to wild-type levels (Fig. 1A; Table 1). It is of interest to note that *P. aeruginosa* PAO1 and ∆*fleQ* demonstrated similar growth behavior under planktonic growth conditions (Fig. 1C), suggesting that the differences in the biofilm architecture are not due to differences in growth rates.

**TABLE 1:** COMSTAT analysis of CSLM images of *P. aeruginosa* biofilms^*b*^

Strain	Total biomass (µm^3^/µm^2^)	Biofilm thickness (µm)
PAO1	6.48 (±3.14)	9.21 (±3.42)
∆*fleQ*	0.13 (±0.17)^*a*^	0.15 (±0.22)^*a*^
∆*fleQ*/pMJT-*fleQ*	5.87 (±3.64)	6.34 (±3.95)
∆*fleQ*/pMJT-*sagS*	0.02 (±0.02)^*a*^	0.02 (±0.02)^*a*^
∆*fleQ*/pMJT-*gcbA*	0.02 (±0.02)^*a*^	0.01 (±0.02)^*a*^
∆*fleQ*/pJN-*bfiR*	0.53 (±0.87)^*a*^	0.03 (±0.03)^*a*^
∆*sagS*	0.38 (±0.38)^*a*^	0.31 (±0.35)^*a*^
∆*sagS*/pMJT-*fleQ*	0.72 (±0.62)^*a*^	0.84 (±0.67)^*a*^

^*a*^ Significantly different from PAO1 or PAO1/pMJT-1, *P* < 0.001, as determined using one-way Anova, followed by Bartlett’s test.

^*b*^ ±, standard deviation.

### FleQ contributes to the antibiotic tolerance phenotype of *P. aeruginosa* biofilms

**Fig 2:**
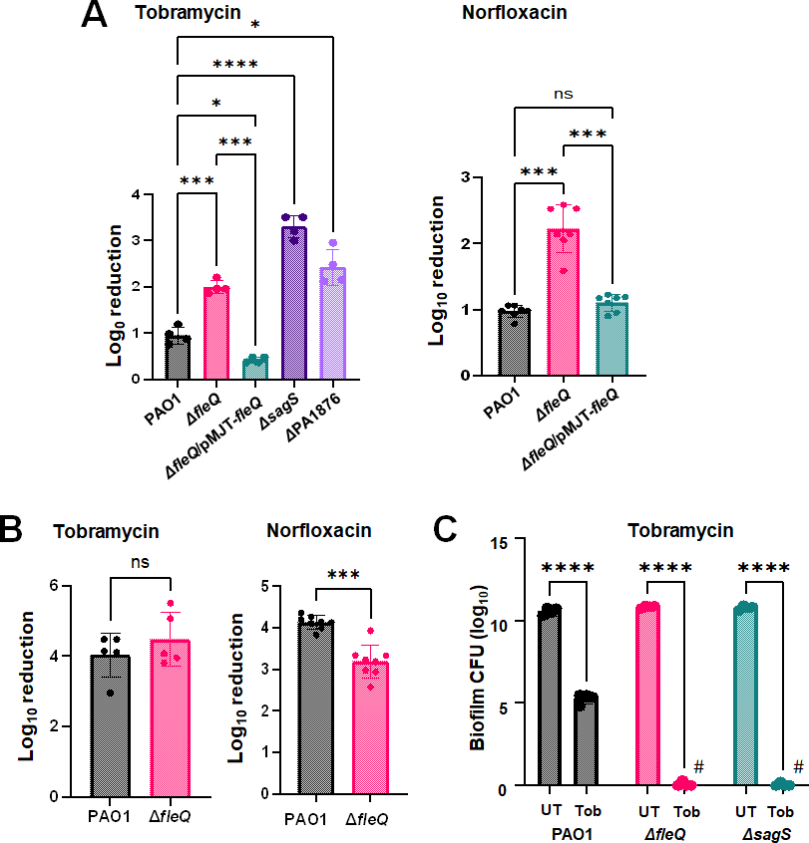
FleQ contributes to the susceptibility phenotype of *P. aeruginosa* biofilms. (**A**) Biofilms of indicated strains were grown in tube reactors under flowing conditions for 3 days and subsequently exposed to tobramycin (150 µg/mL) and norfloxacin (450 µg/mL) for 1 h under flowing conditions. Biofilm susceptibility was determined by log_10_ reduction. *, **, ***, ****, significantly different from the negative control (*P* < 0.05, *P* < 0.001, *P* < 0.0004, *P* < 0.0001, respectively) using ANOVA with Dunnett’s T3 multiple comparisons test. (**B**) *P. aeruginosa* PAO1 and *ΔfleQ* were grown planktonically to exponential phase and then exposed to tobramycin (50 µg/mL) and norfloxacin (50 µg/mL). Susceptibility was determined by log_10_reduction. ***, significantly different (*P* < 0.005) relative to PAO1, as determined using an unpaired *t*-test with Welch’s correction. ns, not significant. (**C**) Biofilm-MBC assays. *P. aeruginosa* PAO1, Δ*fleQ,* and *ΔsagS* were grown as biofilms for 3 days and subsequently treated for 24 h with tobramycin (300 µg/mL) under continuous flowing conditions before recovering and enumerating surviving cells. Biofilm susceptibility to tobramycin was determined by viable counts (biofilm CFU, obtained from biofilm tube reactors having an inner surface area of 25 cm^2^). #, no viable bacteria were detected. ****, significantly different from the untreated biofilm (*P* < 0.0001) using two-way ANOVA with Sidak’s multiple comparisons test. All experiments were carried out at least in triplicate. Error bars indicate standard deviations.

To assess the susceptibility of biofilms formed by ∆*fleQ*, surface-attached cells of wild-type and mutant strains were exposed to 150 µg/mL tobramycin for 1 h under flowing conditions. Biofilms formed by wild-type PAO1 cells demonstrated a less than 1 log reduction in viability following exposure to tobramycin, whereas ∆*fleQ* biofilm cells were reduced by >2.2 logs (Fig. 2A). Multicopy expression of *fleQ* restored the susceptibility phenotype of ∆*fleQ* biofilm cells to tobramycin to wild-type levels (Fig. 2A). It is of interest to note that little to no difference in the susceptibility to antibiotics was noted when grown planktonically (Fig. 2B).

Previous findings indicated a protective role of biofilm matrix components by sequestering positively charged antibiotics such as tobramycin (and colistin) at the biofilm periphery (38). As FleQ contributes to the biofilm matrix by regulating the expression of *pel* and *psl* exopolysaccharides in biofilm (13, 14, 32, 39), we next asked whether the increased susceptibility of ∆*fleQ* biofilms was limited to the positively charged antibiotic tobramycin. We, therefore, evaluated the susceptibility phenotype of biofilms formed by ∆*fleQ* to the neutral fluoroquinolone, norfloxacin. Biofilms formed by ∆*fleQ* were more susceptible to norfloxacin compared to wild-type biofilms (Fig. 2A). Multicopy expression of *fleQ* restored the susceptibility phenotype of ∆*fleQ* biofilm cells to norfloxacin to wild-type levels (Fig. 2A), suggesting the increased susceptibility phenotype of biofilms formed by ∆*fleQ* to be independent of the abundance of exopolysaccharide.

Given the increased susceptibility phenotype of ∆*fleQ* biofilms, we, furthermore, determined whether FleQ affects the resistance of biofilm cells to killing, using a biofilm-MBC assay. *P. aeruginosa* wild-type and Δ*fleQ* cells were grown for 3 days as biofilms, after which time the medium was switched to the same medium containing 300 µg/mL tobramycin. This concentration has been previously demonstrated to eradicate *ΔsagS* but not wild-type biofilm cells (33). Therefore, biofilms by PAO1 and Δ*sagS* were used as controls. Following 24 h of exposure to the antibiotic under flowing conditions, the biofilms were harvested, and the surviving bacteria were enumerated. In agreement with previous findings (33), wild-type PAO1 biofilm cells were not eradicated, whereas no viable cells were recovered from the Δ*sagS* biofilms (Fig. 2C). Inactivation of *fleQ* affected recalcitrance to killing similarly to that of *sagS*, as no surviving cells were recovered from the Δ*fleQ* biofilms following the 24 h treatment with tobramycin (Fig. 2C).

### FleQ does not contribute to biofilm formation in a SagS-dependent manner

The findings above suggested that FleQ contributes to the formation of antibiotic tolerant biofilms. The phenotype was somewhat reminiscent of biofilms formed by the *P. aeruginosa* ∆*sagS* (Fig. 1A and 2A). *sagS* encodes the orphan sensor SagS that has previously been reported to independently promote biofilm development and the activation of biofilm-associated antimicrobial tolerance via the biofilm drug tolerance regulator BrlR (30, 33, 34, 40). BrlR is a global transcriptional regulator, regulating the expression of a multitude of targets including RND pumps and ABC transport systems, with inactivation of *brlR* coinciding with *P. aeruginosa* biofilms being rendered significantly more susceptible to 5 classes of antibiotics. The ABC transport system PA1774-77 has previously been reported to be regulated by BrlR and to contribute to the biofilm susceptibility phenotype to tobramycin as well as norfloxacin (41, 42).

Given the similarity in biofilm architecture and antibiotic susceptibility phenotype between ∆*fleQ* and ∆*sagS* biofilms, we next asked whether FleQ and SagS work synergistically or in concert to affect biofilm formation. We have previously demonstrated that multi-copy expression of certain genes, such as *bfiR* and *brlR*, encoding proteins that function downstream of SagS can restore biofilm phenotypes by ∆*sagS* to wild-type levels (30, 31). Thus, we reasoned that we could use similar epistasis assays to assess the functional relationship between SagS and FleQ. However, while multi-copy expression of *fleQ* restored biofilm formation by ∆*fleQ* to wild-type levels (Fig. 1A), multi-copy expression of *sagS* failed to rescue or enhance biofilm formation by ∆*fleQ* (Fig. 1B; Table 1). Likewise, multi-copy expression of *fleQ* in ∆*sagS* failed to rescue or enhance biofilm formation by the mutant strain (Fig. 1B; Table 1).

**Fig 3:**
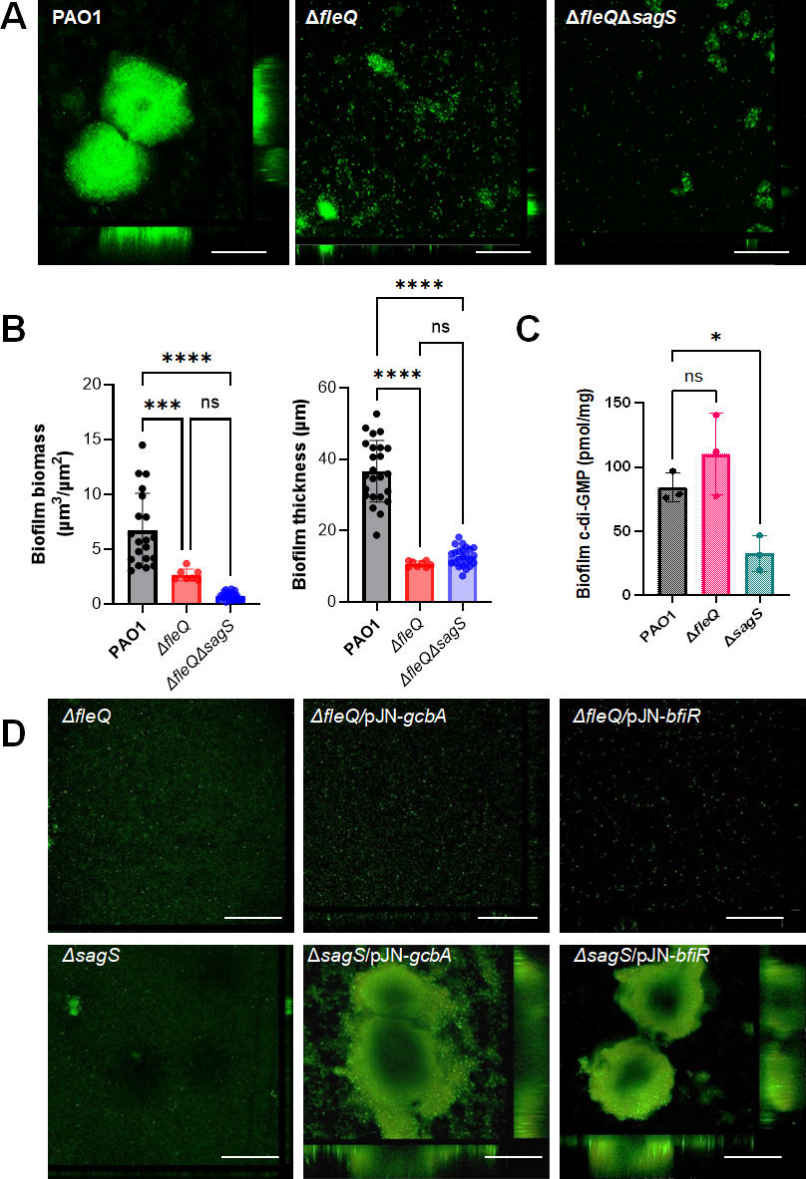
FleQ contributes to biofilm formation in a manner independent of SagS and SagS downstream signaling. (**A**) Representative confocal images of 3-day-old biofilms formed by *P. aeruginosa* PAO1, Δ*fleQ,* and double mutant Δ*fleQΔsagS*. White bar = 100 µm. (**B**) Quantitative of the biofilm biomass and biofilm height by COMSTAT of biofilms formed by *P. aeruginosa* PAO1, Δ*fleQ,* and double mutant ΔfleQΔsagS. **, ***, significantly different (*P* = 0.0001, <0.0001, respectively) relative to PAO1 using ANOVA followed by the Bartlett’s test. Ns, not significant. (**C**) Total cellular c-di-GMP levels in 6-day-old biofilms formed by *P. aeruginosa* PAO1, Δ*fleQ,* and *ΔsagS,* as determined by HPLC quantitative analysis, followed by normalization relative to total cell protein content. *, *P* < 0.05, relative to PAO1 using ANOVA followed by Dunnett’s T3 multiple comparisons test. (**D**) Representative confocal images of 6-day-old *P. aeruginosa* Δ*fleQ* and *ΔsagS* biofilms and mutant biofilms expressing *bfiR* and *gcbA. gcbA* encodes diguanylate cyclase GcbA, *bfiR* the two-component response regulator BfiR. White bar = 100 µm. All experiments were performed in triplicate. Error bars indicate standard deviations.

To discern whether FleQ and SagS to contribute to biofilm formation via distinct or convergent regulatory pathways, we also evaluated biofilm formation by the double mutant ∆*fleQ*∆*sagS*. Biofilms were grown for 3 days in 24-well microtiter plates, and biofilms by wild-type PAO1 and ∆*fleQ* were used as controls. While both *ΔfleQ* and the *ΔfleQΔsagS* double mutation formed biofilms with visibly reduced biofilm biomass than the parent strain PAO1, biofilms by ∆*fleQ*∆*sagS* appeared to be composed of even less biomass than ∆*fleQ* (Fig. 3A). COMSTAT analysis confirmed the overall reduction in biofilm biomass by ∆*fleQ*∆*sagS* relative to ∆*fleQ*; however, the difference was not significant (Fig. 3B). Nevertheless, these findings hinted at FleQ and SagS contributing to biofilm formation synergistically, via distinct, rather than convergent, regulatory pathways.

### FleQ biofilm formation phenotypes cannot be rescued by the downstream targets of SagS

To further assess whether FleQ and SagS contribute to biofilm formation synergistically, we next asked if biofilms by the *fleQ* and *sagS* mutant strains differ in key characteristics such as c-di-GMP levels. FleQ affects biofilm formation via the regulation of biofilm matrix genes by *P. aeruginosa,* specifically the expression of genes contributing to the biosynthesis of Pel and Psl exopolysaccharides (13, 14). In contrast, SagS has been reported to contribute to biofilm formation by linking planktonic-specific Gac/Rsm-dependent signaling and the biofilm-specific TCS BfiSR to facilitate attachment-associated changes in the levels of the small regulatory RNA RsmZ (31, 43, 44) as well as c-di-GMP (30, 35), apparent by ∆*sagS* biofilm cells being characterized by significantly reduced c-di-GMP levels relative to PAO1 biofilm cells. In turn, biofilm formation by ∆*sagS* was restored to wild-type levels by multi-copy expression of *gcbA* encoding the diguanylate cyclase GcbA (30).

In agreement with previous findings (30, 45), ∆*sagS* biofilm cells were found to harbor significantly reduced c-di-GMP levels relative to PAO1 biofilm cells (Fig. 3C). In contrast, ∆*fleQ* biofilms were found to harbor higher levels of c-di-GMP (~100 pmol/mg c-di-GMP) than wild-type biofilms which were found to harbor on average 86 pmol c-di-GMP per mg cell protein (Fig. 3C). The difference in c-di-GMP levels was further supported by multicopy expression of *gcbA* encoding a diguanylate cyclase, restoring biofilm formation by ∆*sagS* to wild-type levels, without having an effect on the architecture of ∆*fleQ* biofilms (Fig. 3D; Table 1). Likewise, while multi-copy expression of *bfiR* encoding the cognate response regulator of the two-component regulatory system BfiSR (23, 31, 43), restored biofilm formation by ∆*sagS* to wild-type levels, expression of *bfiR* had no apparent effect on ∆*fleQ* biofilm formation (Fig. 3D; Table 1). The findings strongly suggest FleQ and SagS contribute to the formation of biofilms via distinct but cooperating mechanisms.

### FleQ works in concert with SagS to enable the formation of antibiotic tolerant biofilms

**Fig 4:**
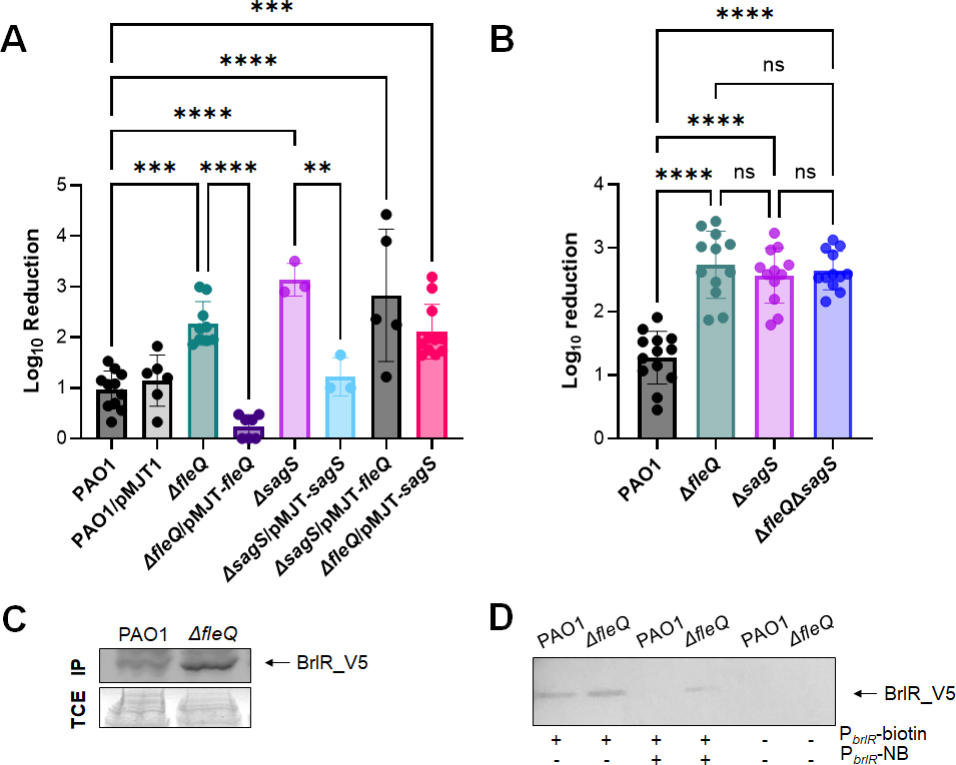
FleQ is required for the tolerance of biofilm cells to tobramycin in parallel with SagS. (**A, B**) Susceptibility phenotype of biofilms grown in tube reactors under flowing conditions for 3 days to tobramycin (150 µg/mL). Susceptibility was determined by log_10_ reduction. **, ***, ****, significantly different from PAO1 biofilms (*P* < 0.005, *P* < 0.001, *P* < 0.0001) using one-way ANOVA, followed by Tukey’s multiple comparison test. ns, not significant. (**C**) Representative immunoblot showing FleQ-dependent abundance of BrlR. Total cell extracts (TCE) obtained from biofilms of indicated *P. aeruginosa* strains expressing a chromosomally located V5/His_6_-tagged BrlR under the control of its own promoter were probed for the presence of BrlR by immunoblot analysis (IB) using anti-V5 antibodies (anti-V5). The corresponding SDS-PAGE gel image obtained post-transfer indicates equal loading. Representative images are shown. (**D**) Representative immunoblot showing FleQ-dependent DNA binding capability of BrlR. BrlR-DNA binding was determined using streptavidin magnetic bead binding assays. Binding assays were carried out using 5 pmol of BrlR-V5/His_6_ protein obtained from the indicated strains and 1 pmol biotinylated P*_brlR_*. Non-biotinylated P*_brlR_* (P*_brlR_*-NB) was used as specific competitor DNA in 20-fold excess. BrlR binding to P*_brlR_* was detected by immunoblot analysis using anti-V5 antibodies. +/−, indicates presence/absence of specific probe or competitor. All experiments were performed in triplicate. Error bars indicate standard deviations

As indicated above, the susceptibility phenotype of ∆*fleQ* biofilms in response to tobramycin and norfloxacin was similar to biofilms formed by *P. aeruginosa* mutant strains ∆*sagS* and ∆PA1876 (Fig. 2A), likely suggesting a role of both FleQ and SagS in the susceptibility phenotype of *P. aeruginosa* biofilms. To determine whether FleQ and SagS contribute to biofilm drug tolerance equally, we also made use of epistasis assays biofilm susceptibility assays in response to tobramycin. Multi-copy expression of *sagS* in Δ*fleQ* failed to restore biofilm susceptibility to tobramycin to wild-type levels, with Δ*fleQ*/pMJT-*sagS* biofilm cells demonstrating CFU reductions exceeding 2.5 logs following exposure to tobramycin for 1 hour (Fig. 4A). Likewise, overexpression of *fleQ* in Δ*sagS* cells did not rescue the biofilm susceptibility phenotype (Fig. 4A). Moreover, the double-mutant ∆*fleQ*∆*sagS* was significantly more susceptible to tobramycin compared to wild-type biofilms, but as susceptible as biofilms formed by the single mutants, Δ*fleQ and* Δ*sagS* (Fig. 4B), strongly suggesting FleQ and SagS to somewhat work in concert.

### Inactivation of *fleQ* does not affect BrlR abundance or BrlR DNA binding

SagS has previously been reported to contribute to the switch from an antimicrobial susceptible to a highly tolerant state by indirectly activating the transcriptional regulator BrlR (30, 33, 34). BrlR, in turn, activates the expression of genes encoding multidrug efflux (MDR) pumps and ABC transporters, conferring tolerance to five classes of antibiotics including tobramycin and norfloxacin (17, 40). As *ΔsagS* biofilms have been characterized by the lack or significantly reduced abundance of active BrlR (30, 34), we first explored if the increased antibiotic susceptibility phenotype of biofilms formed by Δ*fleQ* may be due to decreased abundance of BrlR or alternatively, the presence of non-functional BrlR. Immunoblot analysis suggested the abundance of BrlR to be similar if not somewhat elevated in ∆*fleQ* biofilm cells relative to PAO1 (Fig. 4C). Moreover, using streptavidin pull-down assays and biotinylated P*_blrR_* (17, 36), BrlR produced by ∆*fleQ* biofilm cells was as capable of binding to the *brlR* promoter DNA and outcompeted by non-biotinylated competitor P*_brlR_* DNA that abrogated the binding of BrlR, in a manner similar to BrlR obtained from wild-type biofilms (Fig. 4D).

### FleQ finetunes the expression of a subset of BrlR-regulated genes

**Fig 5:**
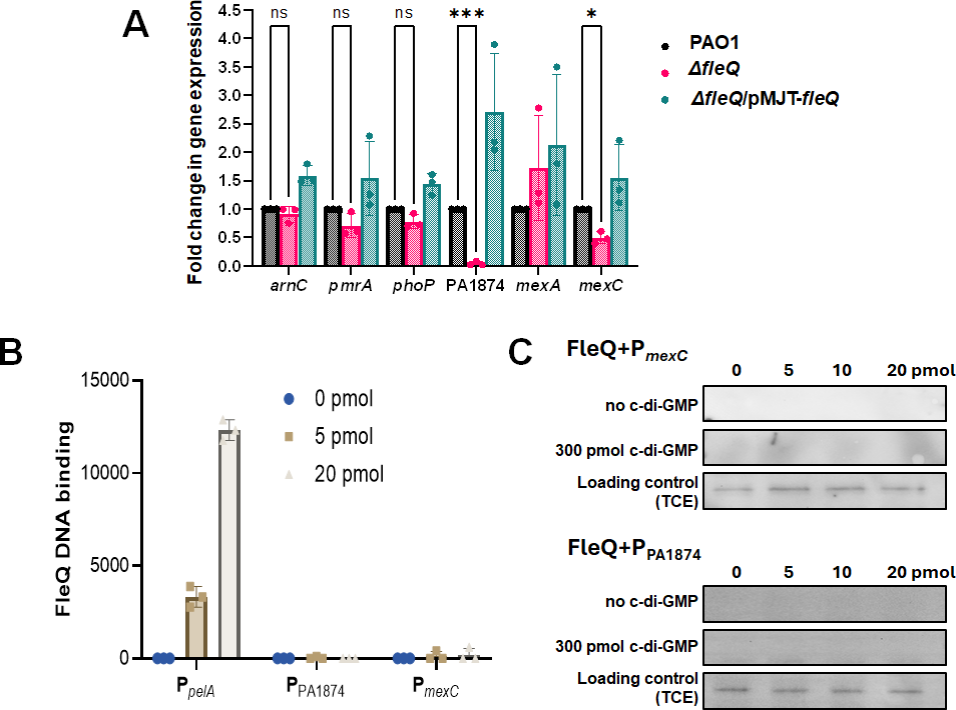
FleQ indirectly affects the expression of a subset of BrlR target genes. (**A**) Transcript abundance of BrlR-target genes in biofilms formed by Δ*fleQ* and complemented Δ*fleQ*/pMJT-*fleQ* mutant strains relative to the wild type (PAO1). Quantitative RT-PCR (qRT-PCR) was carried out using RNA extracted from 3-day biofilms grown in fivefold diluted VBMM. *arnC* encodes a component required for resistance to cationic antimicrobial peptide such as colistin; *mexA* and *mexC* encode components of multi-drug efflux pumps; PA1874 belongs to an operon that encodes ABC transport systems; *phoP* and *pmrA* encode two-component response regulators that control the expression of the *arn* operon. *cysD* was used as the housekeeping gene. Experiments were carried out in triplicate. Error bars indicate standard deviations. *, ***, significantly different relative to the wild-type PAO1 (*P* < 0.5, *P* < 0.0001, respectively) using ordinary two-way ANOVA followed by Dunnett’s multiple comparison test. ns, not significant. (**B, C**) FleQ-DNA binding was determined using streptavidin magnetic bead binding assays, with FleQ binding to the respective promoter regions was detected by immunoblot analysis using anti-V5 antibodies, and subsequent analysis using ImageJ (48). Binding assays were carried out using 100 µg of total biofilm cell extract by PAO1 producing V5-tagged FleQ and 0–20 pmol biotinylated P*_pel_* or P_PA1874_ or *P_mexC_*. (**B**) Quantitative analysis of FleQ DNA binding in the absence of additional c-di-GMP. (**C**) Representative immunoblots of FleQ DNA binding assays in the absence/presence of c-di-GMP and loading controls. Experiments were done in duplicate. Error bars indicate standard deviations.

The above findings underscored that BrlR is both abundant and capable of DNA binding in ∆*fleQ* mutant cells. However, the question remained why biofilms formed by the ∆*fleQ* mutant strain, despite harboring functional BrlR at elevated abundance relative to wild-type biofilms, were susceptible to tobramycin and norfloxacin. We, therefore, asked if FleQ may somehow affect the functionality of BrlR, by assessing whether BrlR downstream targets are expressed, using quantitative reverse transcriptase PCR (qRT-PCR). Target genes included genes encoding Mex efflux pumps (*mexA, mexC*) (17), the ABC transporter PA1874-77 (41), and genes linked to colistin resistance, namely, *pmrA*, *phoP,* and *arnC* (46, 47). Inactivation or multicopy expression of *fleQ* had little to no effect on the BrlR target *mexA,* as well as genes linked to colistin resistance (*pmrA*, *phoP, arnC*) relative to wild-type biofilms (Fig. 5A). In contrast, however, significant differences in the transcript abundance were noted for the BrlR target PA1874, and somewhat for *mexC* in biofilms inactivated in or overexpressing *fleQ* relative to wild-type biofilms (Fig. 5A), indicating FleQ to finetune the expression of a subset of genes under regulatory control by BrlR previously linked to biofilm antibiotic tolerance.

To determine if FleQ directly affected the expression of *mexC* and PA1874, we determined whether FleQ is capable of binding to the promoter of the PA1874-77 and *mexCD-oprJ* operons. FleQ binding to the *pel* promoter was used as a control. While FleQ was capable of binding to the *pel* promoter (Fig. 5B) as previously reported (14), no binding to the promoter region of PA1874-77 and *mexCD-oprJ* was noted, neither in the absence nor in the presence of c-di-GMP (Fig. 5B and C), excluding a direct link between FleQ, PA1874-77, and *mexCD-oprJ*. This was further supported by the absence of a possible FleQ binding site in the promoter region of PA1874-77 and *mexCD-oprJ* (14, 49).

### FleQ interacts with the orphan sensor SagS

**Fig 6:**
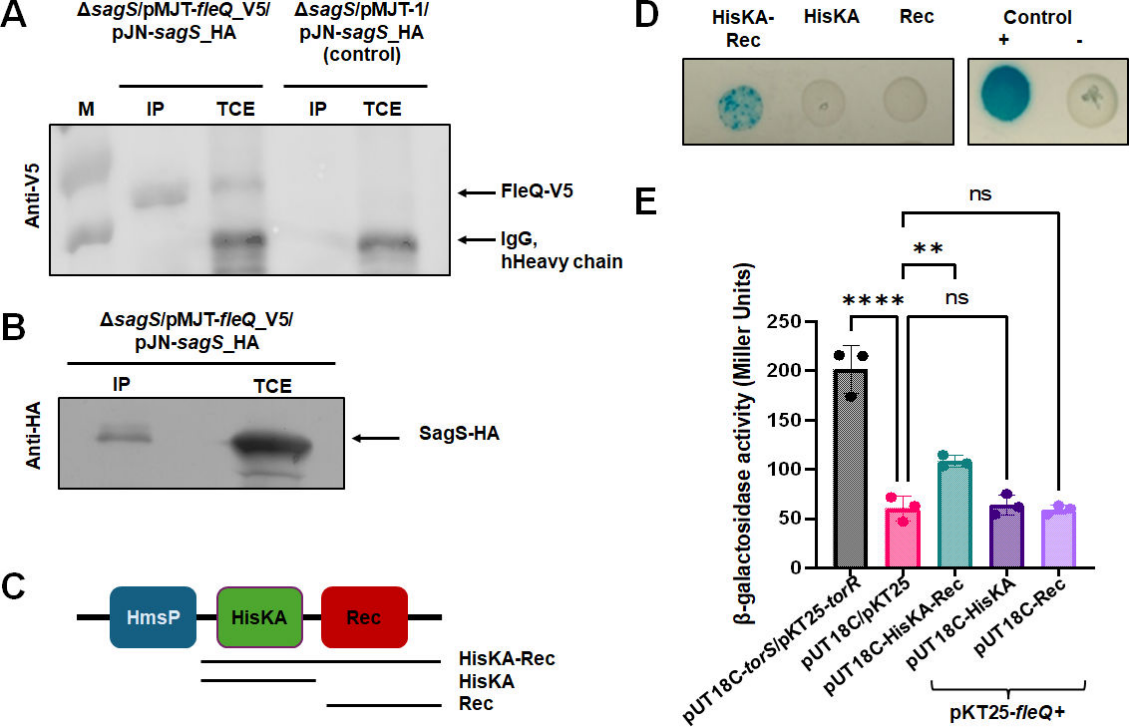
FleQ interacts with SagS via its HisKA-Rec domain. (**A**) Representative image of immunoblot showing the abundance of FleQ in total cell extracts (TCE) and in pulldowns (IP) using SagS as prey. Strain *ΔsagS/*pMJT*-*1*/pJN-sagS* was used as control. M, protein ladder. (**B**) Representative image of immunoblot showing the abundance of SagS in total cell extracts (TCE) and in pulldowns (IP) using FleQ as prey. (**C**) Overview of SagS domains and SagS domain constructs. Lines underneath the domains indicate the composition of the SagS domain constructs, while the names of the resulting constructs are given next to the lines. (**D**) Representative images of bacterial adenylate cyclase two-hybrid assay (BACTH) results. DHM1/pUT18C-*torS*/pKT25-TorR was used as the positive control (+) and DHM1/pUT18C/pKT25 was used as the negative control (−). Bluish-green coloration represents a cleavage of X-gal by β-galactosidase indicative of protein-protein interaction. (**E**) Quantitative analysis of β-galactosidase activity using the Miller assay (51) of DHM1 strains co-expressing *fleQ* (pKT25-*fleQ*) and SagS-domain constructs (pUT18C -HisKA-Rec, pUT18C-HisKA, or pUT18C-HisKA-Rec). *E. coli* strains DHM1/pUT18C-*torS*/pKT25-*torR* and DHM1/pUT18C/pKT25 were used as controls. Experiments were done in triplicate. Error bars represent standard deviation. ** and ****, significantly different from the negative control (*P* < 0.01, *P* < 0.0001, respectively) using ANOVA followed by Dunnett’s T3 multiple comparisons test.

The above findings indicated FleQ to not affect *brlR* expression or BrlR DNA binding, but instead to indirectly finetune the expression of the BrlR regulated genes PA1874-77 and *mexCD-oprJ*. However, this raised the question of how FleQ accomplishes this. As SagS has been described as a regulatory hub, connecting multiple overlapping signaling system that only indirectly modulates *brlR* expression to enable biofilm drug tolerance (30, 45, 50)*,* we, therefore, asked whether FleQ interacts with SagS. To probe for interactions between FleQ and SagS, we made use of co-immunoprecipitation. Full-length FleQ was found to co-purify with SagS when HA-tagged SagS was used as bait proteins (Fig. 6A), while no FleQ was detected in the controls (Fig. 6A), confirming the specificity of the interaction between FleQ and SagS. This was further confirmed by the detection of SagS when V5-tagged FleQ was used as bait (Fig. 6B).

Previous findings indicated the switch function by SagS to be due to the modular composition of SagS (30) (Fig. 6C), with the cytoplasmic HisKA-Rec domains being required for its function (25, 30, 45). We, therefore, asked if FleQ interacts with SagS via one or both of its cytoplasmic domains, using bacterial adenylate cyclase‐based two‐hybrid (BACTH) assay in *E. coli*. Full-length FleQ and truncated SagS domain constructs, harboring only the HisKA-Rec, HisKA, or Rec domains of SagS (Fig. 6C), were fused to either the T18 or the T25 subunit of adenylate cyclase and then co-expressed in *E. coli* DHM1 strain to test for interaction. For controls, we made use of TorR and TorS (52), as well as a control strain harboring empty vectors (pKT25, pUT18C) (Fig. 6D), with the positive control being detected as blue colonies on medium containing X-Gal (5-bromo-4-chloro-3-indolyl-β-D-galactopyranoside) and the negative control coinciding with no color change (Fig. 6D). A positive interaction was noted between FleQ and the SagS construct harboring the HisKA and Rec domain (Fig. 6D). In contrast, little to no interactions were noticed between FleQ and the SagS constructs only harboring the HisKA or the Rec domain (Fig. 6D). Visual observations of protein-protein interactions were confirmed by Miller assays (Fig. 6E), suggesting FleQ to primarily interact with SagS via its HisKA-Rec domains.

## DISCUSSION

The goal of this study was to explore the role of FleQ in the formation of drug tolerant biofilms and to determine whether FleQ intersects with other regulatory systems to enable the transition from the planktonic to the biofilm mode of growth.

We demonstrated that prolonged growth of ∆*fleQ* cells at the surface results in the formation of biofilms demonstrating severely reduced biomass and lack of complex three-dimensional architecture, including biofilm cell aggregates and microcolonies (Fig. 1). The resulting biofilms were highly susceptible to two antibiotics, the neutral norfloxacin and the positively charged tobramycin (Fig. 2A), with inactivation of *fleQ* disabling the recalcitrance of *P. aeruginosa* biofilms to tobramycin (Fig. 2C). Thus, while FleQ has been known to contribute to biofilm formation by expressing biofilm genes in a manner dependent on the levels of c-di-GMP (37), our findings expand on the role of FleQ from regulating the transition to the biofilm mode of growth to FleQ contributing to the antibiotic tolerance phenotype of biofilms. This was further supported by FleQ modulating the expression of *mexC* and PA1874 (Fig. 5A). Previous findings indicated that complete deletion of the genes encoding this pump, PA1874 to PA1877 (PA1874-1877), in both *P. aeruginosa* PA14 and PAO1 resulted in an increase in sensitivity to various antibiotics including tobramycin, gentamicin, norfloxacin, and ciprofloxacin, specifically when this mutant strain is growing as a biofilm (41, 42) (Fig. 2A). Moreover, inactivation of the respective pump has been shown to eliminate the recalcitrance of biofilms to killing by tobramycin (41, 42), in a manner similar to inactivation of *fleQ* (Fig. 2C).

**Fig 7:**
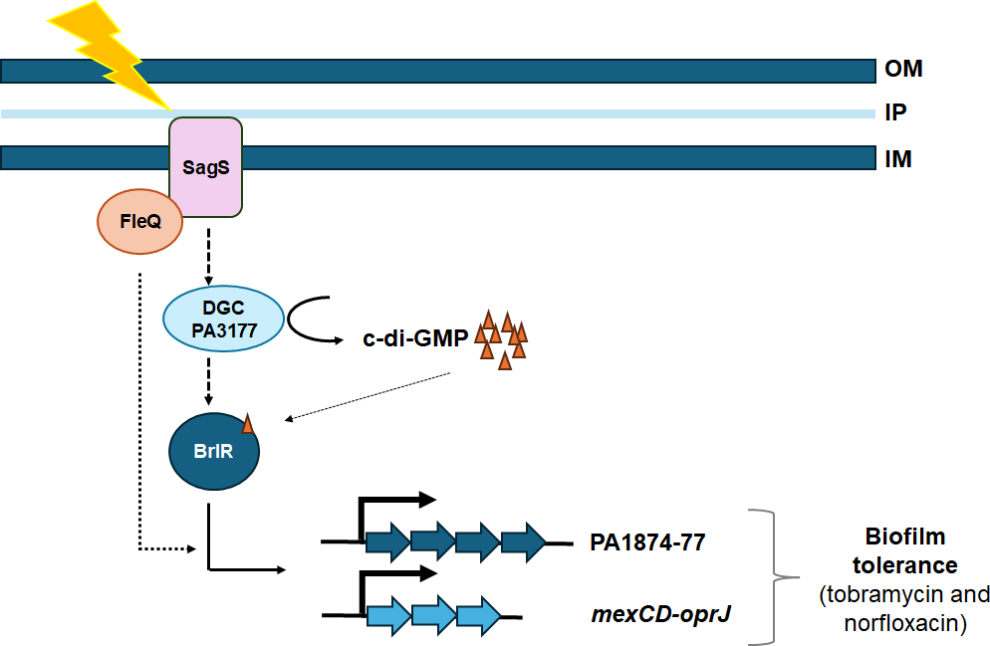
Model of FleQ finetuning BrlR target gene expression to modulate biofilm antibiotic tolerance. SagS indirectly contribute to the abundance and activation of the c-di-GMP responsive transcriptional regulator BrlR (36), likely by SagS contributing to the pool of available c-di-GMP generated by the diguanylate cyclase (DGC) PA3177 (33, 35). FleQ does not affect the abundance of BrlR or its DNA binding capability but indirectly affects the expression of a subset of BrlR target genes (17, 41). Dashed line, indirect effect of SagS on BrlR; dotted line, indirect effect of FleQ on BrlR, resulting in finetuning the expression of a subset of BrlR target genes.

PA1874-77 has previously been shown to be under the control of the transcriptional regulator BrlR (17, 40). However, we found no evidence of FleQ affecting BrlR abundance or BrlR-DNA binding (Fig. 4C and D), nor were we able to demonstrate FleQ directly regulating the expression of the PA1874-77 operon (nor *mexCD-oprJ*) (Fig. 5B). However, the expression of *brlR*, and in turn PA1874-77, has been shown to be dependent on SagS (30, 45). SagS and FleQ functioning synergistically to enable the formation of inherently antibiotic tolerant *P. aeruginosa* biofilms was apparent by epistasis assays (Fig. 4A), with multicopy expression of *sagS* failing to restore biofilm antibiotic tolerance by *ΔfleQ* to wild-type levels (and *vice versa*), and by biofilms formed by the *ΔfleQΔsagS* double mutant being as susceptible as *ΔfleQ* and *ΔsagS* biofilms (Fig. 4B). Given the synergy between FleQ and SagS and considering that SagS activates *brlR* while FleQ modulates the expression of BrlR target genes, the finding suggests FleQ contributes to biofilm tolerance by finetuning BrlR (upon activation in a SagS-dependent manner) (Fig. 7), apparent by FleQ only modulating a subset of BrlR downstream targets, specifically the PA1874-77 operon (Fig. 5A).

While our findings failed to provide a direct link between FleQ and the regulation of *mexCD-oprJ* and PA1874-77, our findings indicate a close association between FleQ and SagS, with FleQ interacting with SagS via its HisKA-Rec domains (Fig. 6). The findings likely hint of the signaling network by the dual sensory switch protein SagS to comprise FleQ (or alternatively, the regulatory network of FleQ comprising SagS), with the two proteins affecting BrlR function, thus finetuning the BrlR regulon, to enable the formation of inherently tolerant *P. aeruginosa* biofilms (Fig. 7). However, the importance of the interaction between FleQ and SagS, and whether the two proteins need to interact for FleQ to finetune gene expression, is unknown and will be the focus of future studies.

## MATERIALS AND METHODS

### Bacterial strain, media, and growth conditions

*P. aeruginosa* PAO1 and indicated mutant strains used in this study are listed in Table 2. All planktonic cultures were grown in Lennox Broth (LB, BD Biosciences) in flasks at 220 rpm and 37°C. Biofilms were grown as indicated below. Antibiotics for plasmid maintenance were used at the following concentrations: 250 µg/mL carbenicillin (Carb) and 50–75 µg/mL gentamicin (Gm) for *P. aeruginosa* and 100 µg/mL ampicillin (Amp), 25–50 µg/mL kanamycin (Km), and 20 µg/mL gentamicin for *Escherichia coli*. Arabinose (0.1%) was added to induce the expression of genes cloned under the control of the arabinose inducible P_BAD_ promoter.

**TABLE 2:** Bacterial strains and plasmids

Strains/plasmids	Relevant genotype description	Source
Strains		
*Escherichia coli*		
DH5α	F^−^, φ80*lacZ*ΔM15, Δ(*lac*ZYA-*argF*)U169, *recA*1, *endA*1 *hsdR*17(rk^−^, mk^+^), *pho*A, *sup*E44 λ^−^*thi*-1, *gyr*A96, *rel*A.1	Invitrogen corp.
DHM1	F^−^, *cya-854*, *rec*A1, *endA*1, *gyrA96* (*Nal^r^*), *thi1*, *hsdR*17, *spoT1*, *rfbD1*, *glnV44(AS*)	(53)
*Pseudomonas aeruginosa*		
PAO1	Wild-type strain PAO1	B.H. Holloway
Δ*fleQ*	PAO1; Δ*fleQ*	(32)
Δ*sagS*	PAO1; Δ*sagS* (PA2824)	(31)
Δ*fleQ*ΔsagS	PAO1; Δ*fleQ*Δ*sagS*	This study
ΔPA1876	PAO1, PA1876::IS*phoA*; Tet^R^	(54)
Plasmids		
pJN105	Arabinose-inducible expression vector; pBRR-1 MCS; *araC*-P_BAD_; Gm^R^	(55)
pJN-*sagS*	C-terminal HA-tagged *sagS* cloned into pJN105 vector between Nhe1 and Sac1 restriction site; Gm^R^	(31)
pMJT-1	Arabinose-inducible expression vector; cassette of pJN105 cloned into pUCP18; *araC*-P_BAD_; Amp^R^, Carb^R^	(56)
pMJT-V5-*fleQ*	N-terminal V5-tagged *fleQ* cloned into pMJT-1 vector between Nhe1 and Sac1 restriction sites; Carb^R^	This study
pMJT-*sagS*	C-terminally HA-tagged *sagS* cloned into pMJT1 at NheI/SacI; Carb^R^	(31)
pJN-*bfiR*	C-terminally V5/6xHis-tagged *bfiR* cloned into pJN105 at EcoRI/SpeI; Gm^R^	(43)
pMJT-*gcbA*	pMJT-PA4843; *gcbA* cloned into pMJT-1 at XbaI; Carb^R^	(57)
pMJT-*brlR*	C-terminally His_6_/V5-tagged *brlR* cloned into pMJT1; Carb^R^	(40)
pKT25	Expression vector harboring the T25 fragment of CyaA; Km^R^	(53)
pKT25-*torR*	pKT25 harboring TorR; Km^R^	(52)
pKT25-*fleQ*	*fleQ* cloned between the Xba1 and Sma1 restriction site, downstream of the T25 fragment; Km^R^	This study
pUT18C	Expression vector harboring the T18 fragment of CyaA; Amp^R^	(53)
pUT18C-*torS*	pUT18C harboring the Hpt domain of TorS; Amp^R^	(52)
		
pUT18C-sagS_HisKA-Rec	pUT18C harboring SagS HisKA-Rec domain; Amp^R^	This study
pUT18C-sagS_HisKA	pUT18C harboring SagS HisKA domain; Amp^R^	This study
pUT18C-sagS_Rec	pUT18C harboring SagS Rec domain; Amp^R^	This study
CTX-P*brlR-brlR*-His_6_V5	pMini-CTX harboring C-terminal His_6_/V5-tagged *brlR* under the control of the *brlR* promoter region (1–120 bp upstream of the *brlR* start codon); Tet^R^	(34)
pRK2013	Helper plasmid for triparental mating; *mob; tra*; Km^R^	(58)
pEX18Gm	Allelic replacement suicide vector; pUC18 MCS; *oriT^+^*; *sacB^+^*; Gm^R^	(59)
pEX-*sagS*	Allelic replacement vector for *sagS*	(31)

### Strain construction

Isogenic mutants were constructed by allelic replacement using sucrose counterselection, as previously described (60), with the gene replacement vector pEX18Gm (59). Complementation and overexpression were accomplished by placing the respective genes under the control of an arabinose inducible promoter in the pJN105 or pMJT-1 vector (Table 2). Note that plasmid-borne *fleQ* harbored a V5-tag while *sagS* harbored an HA-tag (31). The V5-tag was introduced into *fleQ* via PCR amplification, using the primers and restriction sites indicated in Table 3. Plasmids were introduced via conjugation or electroporation. For bacterial two-hybrid assays, full length FleQ and SagS domain constructs (HiusKA, HisKA-Rec, Rec) were amplified using primers listed in Table 3 and cloned in frame into pKT25 and pUT18C plasmids. The generated recombinant plasmids (pKT25-*fleQ*, pUT18C-*sagS,* and pUT18C-HisKA-Rec) were subsequently introduced into *E. coli* DHMI strains by chemical transformation. All constructs were verified via DNA sequencing using primers listed in Table 3.

**TABLE 3:** Oligonucleotides

Oligonucleotides	Sequences^*a*^^,*b*,*c*^
Deletion	
sagSdel_for1_EcoRI	GCGCGCGCgaattcGCTGCAGGTAGCCGCTAC
sagSdel_rev1_BamHI	GCGCGCGCggatccCTCCGCATCGATGTTGTAGG
sagSdel_for2_BamHI	GCGCGCGCggatccGAGGAGCATCCCATCGACC
sagSdel_rev2_HindIII	GCGCGCGCaagcttGCCAGTCACGATGAGCGG
Cloning into pMJT-1	
V5-*fleQ*-NheI-for	GCGCGCGCgctagcATG**ggtaagcctatccctaaccctctcctcggtctcgattctacg** ATGTGGCGCGAAACCAAACTC
*fleQ*-SacI-rev	GCGCGCGCgagctcTCAATCATCCGACAGGTCGTC
Cloning into pKT25	
linker-Xba1-*fleQ*-F	GCGCGCGCtctagaAATGTGGCGCGAACCAAACTC
linker-Sma1-*fleQ*-R	GCGCGCGCcccgggTCAATCATCCGACAGGTCGTC
Cloning into pUT18C	
sagS_HisKA-Rec-F	GCGCGCGCggatccGATGCTCGGGCGCATCTCGGTAG
SagS_HisKA-Rec-R	GCGCGCGCgaattcCTAGTCGCTCGCGGTGAGCGG
sagS_HisKA-F	GCGCGCGCggatccGATGCTCGGGCGCATCTCGGTAG
SagS_HisKA-R	GCGCGCGCgaattcCTAGCGCTGTTCTCCCGTGGTG
SagS_Rec-F	GCGCGCggatccGATGGTCAGCCCTCCGCTGCAG
SagS_Rec-R	GCGCGCGCgaattcCTAGTCGCTCGCGGTGAGCGG
Streptavidin bead DNA binding assay	
P_*pelA*-F^*c*^	TTCCTCGCACGCAACTGAAG
P-*pelA*-R2	TCGGAAGCATGGATGCGCTTTC
P_PA1874_for	GAGGATCGGTTGGATAAC
P_PA1874_rev^*c*^	CGACATTTCCGACATTCC
PbrlR-120F^*c*^	ATGGGTTCACTGTTGGGAC
PbrlR-120R	GGTTCCTGCTGCGGGATAC
P_mexC_F^*c*^	GTGCGCCTTCGCCGTG
P_mexC_R	GACACACCCGACCGTTG
Sequencing primers	
pUT18C_MCS_For	GCCTGTTCGACGATGGG
pUT18C_MCS_Rev	CTCTGACACATGCAGCTC
pKT25_MCS_For	CGACATGTTCGCCATTATGC
pKT25_MCS_Rev	CGGGCCTCTTCGCTATTAC
sagS_deletion_check_rev	GCAGTACCTGGTCATGCTGG
sagS_deletion_check_for	CGATCTCGATCAGGCGCAG
qRT-PCR primers	
*cysD*_FOR-qPCR	CTGGACATCTGGCAATACAT
*cysD*_REV-qPCR	TCTCTTCGTCAGAGAGATGC
*arnC*-(PA3553)_RT-FOR	GAACGCAGTACCTTCATCC
*arnC*-(PA3553)_RT-REV	GGTCGAACATCAGGTTGAG
*mexA*(PA0425)_RTFOR	ACCCTGAATACCGAGCTG
*mexA*(PA0425)_RTREV	GGTCGATCTGGTAGAGCTG
*mexC*(PA4599)_RTFOR	CCTGCTGTTCCAGATCG
*mexC*(PA4599)_RTREV	CGGTATCGAAGTCCTGCT
*pmrA*-RT-F	GAGAATACTGCTGGCCGAGGA
*pmrA*-RT-R	GTCCGATGTCGAGCACCAG
PA1874-RT-F	GTCGATCCAGGCGAAAGTTA
PA1874-RT-R	CGAACCGCTCTTCGAGTATC
*phoP*RT-for	GTCAGCGAATACCACCAC
*phoP*RT-rev	GCTTCCAGTTCCTCGAAC

^*a*^ Restriction sites are indicated in lower case.

^*b*^ V5 tag sequences are written in bold and in lower case.

^*c*^ Primers were biotinylated.

### Planktonic growth curve

Overnight cultures of PAO1, Δ*fleQ,* and Δ*fleQ*/pMJT-V5-*fleQ* were inoculated as 1% inoculum into fresh VBMM with or without Carb (250 µg/mL). The optical density (OD) was measured every hour at 600 nm.

### Biofilm growth

*P. aeruginosa* biofilms were grown in fivefold diluted VBMM medium using a continuous flow tube reactor system (1 m long size 13 or 14 silicone tubing, Masterflex, Cole Parmer, Inc.) at a flow rate of 0.1 mL/min or 0.2 mL/min, respectively, as well as in flow cells (BioSurface Technologies) at a flow rate of 0.2 mL/min (61–63). Following 6 days of growth, the biofilms were subjected to microscopy analysis or antimicrobial susceptibility testing as described below. For plasmid maintenance, gentamicin and carbenicillin were used in tube reactors at a concentration of 2 µg/mL.

### Biofilm architecture analysis

Architecture of biofilms grown in flow cells was assessed via confocal laser scanning microscopy (CLSM). CSLM was carried out using a Leica TCS SP5 confocal microscope. Prior to confocal microscopy, biofilms were stained using the *Bac*Light LIVE/DEAD viability stain (Life Technologies) at a 1/1,000 dilution in the growth medium, with 2 mL of the working *Bac*Light LIVE/DEAD solution used per flow cell. The CLSM images were processed using LAS AF software v2.4.1. Quantitative analysis of the images was performed using COMSTAT MATLAB package and COMSTAT2 (64, 65).

### Antibiotic susceptibility assays

All biofilms subjected to antimicrobial susceptibility assays were grown in tube reactors. Biofilms grown for 3 days were treated for 1 h with tobramycin (150 µg/mL) and norfloxacin (450 µg/mL). Following exposure of biofilms to the respective antimicrobial agents under flowing conditions, biofilms were harvested from tube reactors by squeezing the tubing, followed by the extrusion of the cell paste as previously described (63). To ensure complete disaggregation of cell aggregates, the resulting suspension was first homogenized for 15 s using a Biospec tissue tearer. The cell suspension was then serially diluted and spread plated onto LB agar. Viability was determined via CFU counts. Susceptibility is expressed as log_10_ reduction in viability.

Biofilm-MBC is defined as the concentration of antimicrobial where no additional killing is observed (17, 33, 40). We have previously shown that *P. aeruginosa* PAO1 biofilms demonstrate no significant increase in log reduction at concentrations greater than approximately 75 µg/mL of tobramycin after 24 h of treatment (17, 33, 40). Biofilms used for MBC assays were grown in continuous-flow tube reactors as described above for 3 days and subsequently exposed for 24 h to medium containing 300 µg/mL tobramycin. Following 24 h of treatment, biofilms were harvested, homogenized, and serially diluted. CFUs were determined via viable counts.

For planktonic susceptibility assays, overnight cultures of indicated *P. aeruginosa* strains were inoculated into fresh LB at 1% and grown for 3 h at 37ᵒC, at which point, the cells were treated for 30 min with tobramycin (50 µg/mL) or norfloxacin (50 µg/mL). Treated and untreated control cells were harvested by centrifugation and serially diluted and spread plated as described above.

### Quantification of c-di-GMP levels

c-di-GMP was extracted in triplicate from 6-day biofilms of wild-type and mutant strains using heat and ethanol precipitation and quantitated essentially as described (66, 67). Briefly, c-di-GMP was extracted in triplicate using heat and ethanol precipitation followed. Supernatants were combined, dried using a Speed-Vac, and resuspended in 10 mM ammonium acetate. Samples (20 µL) were analyzed using an Agilent 1100 HPLC equipped with an autosampler, degasser, and detector set to 253 nm and separated using a reverse-phase C_18_ Targa column (2.1 × 40 mm; 5 µm) at a flow rate of 0.2 mL/min with the following gradient: 0–9 min, 1% B; 9–14 min, 15% B; 14–19 min, 25% B; 19–26 min, 90% B; 26–40 min, 1% B (buffer A, 10 mM ammonium acetate; buffer B, methanol plus 10 mM ammonium acetate). Commercially available cyclic di-GMP was used as a reference for the identification and quantification of cyclic di-GMP in cell extracts.

### Immunoblot analysis and co-immunoprecipitation pull-downs

Co-immunoprecipitation pull-down assays were used to assess the interactions between SagS and FleQ in total protein cell extracts of cells co-producing V5-tagged FleQ and HA-tagged SagS. Pull-down assays were carried out using 2 mg of total cellular protein extracts. Following immunoprecipitation of V5-tagged proteins using immobilized anti-V5 antibodies at a concentration of 2 µg/mL, immunoprecipitation eluates were separated by SDS-PAGE and assessed by immunoblot analysis for the presence of HA-tagged prey SagS protein using anti-HA antibodies. Similarly, immunoprecipitation of HA-tagged protein was carried out using immobilized anti-HA antibodies, with the presence of V5-tagged proteins probed using anti-V5 antibodies. Densitometry of immunoblot bands was performed using the Gels Analysis tool of the ImageJ program (48).

### Bacterial adenylate cyclase two-hybrid assays

Protein-protein interactions were assessed by the bacterial adenylate cyclase two-hybrid (BACTH) as previously described (68, 69). Proteins of interest were fused to the T18 or T25 fragment of *Bordetella pertussis* adenylate cyclase. The recombinant plasmids were co-transformed into *E. coli* DHM1 strain lacking the adenylate cyclase gene *cya*. Interaction of proteins of interest leads to heterodimerization of the T18 and T25 fragments, with interaction between the two hybrid proteins reconstituting the catalytic domain of adenylate cyclase, leading to cyclic AMP (cAMP) synthesis and transcription of the *lac* operon. DHM1 transformants were OD-adjusted to 0.1, and 2 µl per dilution spotted onto LB agar containing 50 µg/mL ampicillin, 50 µg/mL kanamycin, X-Gal (0.1 mM), and IPTG (isopropyl-β-D-thiogalactopyranoside, 0.1 mM). Plates were incubated at 30°C for up to 48 h, and colonies were examined for blue coloration. TorR and TorS protein served as positive controls (52), and the empty plasmids acted as the negative control. The interactions were quantitatively analyzed using β-galactosidase activity assays (51).

### RNA isolation and qRT-PCR

Cells used for RNA isolation and qRT-PCR were grown as biofilms in fivefold diluted VBMM minimum media supplemented with carbenicillin (2 µg/mL) or gentamycin (2 µg/mL) in tube reactors for 3 days as described above. Biofilms were harvested into 3 mL RNAProtect, vortexed, and treated with 400 µg/mL lysozyme for 5 min prior to RNA isolation by the E.Z.N.A. Total RNA Kit I (OmegaBiotek). Prior to cDNA synthesis using the iScript cDNA synthesis kit (BioRad), RNA (1 µg) was DNAse treated using the TurboDNase-free (Invitrogen). qRT-PCR was carried out using BioRad CFX Connect Real-Time PCR Detection System and SsoAdvanced SYBR Green Supermix (BioRad), along with primers listed in Table 3. The housekeeping gene *cysD* was used. Relative transcript abundance was calculated using the CFX Manager Software (BioRad), by first normalizing the threshold cycle value (CT) to *cysD*, and then determining transcript abundance ratios. Expression of *brlR* downstream genes was determined using the 2^−ΔΔCT^method (70). Melting curve analyses were employed to verify specific single product amplification. The qRT-PCR analysis was performed using three biological triplicates.

### Streptavidin magnetic bead DNA binding assay

DNA binding by BrlR and FleQ was assessed using the streptavidin magnetic bead DNA binding assay as previously described (60). Biotinylated target DNA fragments (P*brlR*, P*_fleQ_,* PA1874*,* P*_mexC_*) were amplified using the primer pairs listed in Table 3. A total of 0–20 pmol of biotinylated target DNA was incubated for 30 min at room temperature with biofilm total cell extract containing 25 pmol of His_6_V5-tagged BrlR or 100 µg of V5-tagged FleQ (as indicated by immunoblot analysis using purified His_6_V5-tagged BrlR or biofilm total cell extract containing V5-tagged FleQ) in 25 mM Tris-Cl, pH 8, 5 mM MgCl_2_, 0.5 mM dithiothreitol, 1 mM EDTA, and 50 ng/ul poly(dI-dC). For specific competition, non-biotinylated P*brlR* target DNA (0–50 pmol) was used. c-di-GMP (300 pmol) was added where indicated. Briefly, streptavidin magnetic beads (Thermo Scientific, 100 µg) were used to capture biotinylated DNA. Following three washes, the proteins co-purified with the biotinylated DNA were separated by 10% SDS/PAGE and assessed by immunoblot analysis for the presence of BrlR or FleQ using anti-V5 antibodies (Invitrogen). An aliquot prior to the addition of streptavidin magnetic beads was used to determine total BrlR or FleQ present in each DNA binding assay (loading control). Bands were visualized by chemiluminescence.

### Statistical analysis

For pairwise comparison, a two-tailed Student’s *t*-test assuming equal variance or using single-factor analysis of variance (ANOVA) was used. Unless otherwise noted, all experiments were performed at least in triplicate using biological replicates.

## References

[1] Sauer K, Stoodley P, Goeres DM, Hall-Stoodley L, Burmølle M, Stewart PS, Bjarnsholt T. 2022. The biofilm life cycle: expanding the conceptual model of biofilm formation. Nat Rev Microbiol 20:608–620. doi:10.1038/s41579-022-00767-0 35922483 PMC9841534

[2] Davies D. 2003. Understanding biofilm resistance to antibacterial agents. Nat Rev Drug Discov 2:114–122. doi:10.1038/nrd1008 12563302

[3] Costerton JW, Cheng KJ, Geesey GG, Ladd TI, Nickel JC, Dasgupta M, Marrie TJ. 1987. Bacterial biofilms in nature and disease. Annu Rev Microbiol 41:435–464. doi:10.1146/annurev.mi.41.100187.002251 3318676

[4] Costerton JW, Stewart PS, Greenberg EP. 1999. Bacterial biofilms: a common cause of persistent infections. Science 284:1318–1322. doi:10.1126/science.284.5418.1318 10334980

[5] Ruiz M, Ewig S, Torres A, Arancibia F, Marco F, Mensa J, Sanchez M, Martinez JA. 1999. Severe community-acquired pneumonia. Risk factors and follow-up epidemiology. Am J Respir Crit Care Med 160:923–929. doi:10.1164/ajrccm.160.3.9901107 10471620

[6] Sadikot RT, Blackwell TS, Christman JW, Prince AS. 2005. Pathogen-host interactions in Pseudomonas aeruginosa pneumonia. Am J Respir Crit Care Med 171:1209–1223. doi:10.1164/rccm.200408-1044SO 15695491 PMC2718459

[7] Cole SJ, Records AR, Orr MW, Linden SB, Lee VT. 2014. Catheter-associated urinary tract infection by Pseudomonas aeruginosa is mediated by exopolysaccharide-independent biofilms. Infect Immun 82:2048–2058. doi:10.1128/IAI.01652-14 24595142 PMC3993445

[8] Restrepo MI, Babu BL, Reyes LF, Chalmers JD, Soni NJ, Sibila O, Faverio P, Cilloniz C, Rodriguez-Cintron W, Aliberti S, GLIMP. 2018. Burden and risk factors for Pseudomonas aeruginosa community-acquired pneumonia: a multinational point prevalence study of hospitalised patients. Eur Respir J 52:1701190. doi:10.1183/13993003.01190-2017 29976651

[9] Wagner VE, Iglewski BH. 2008. P. aeruginosa biofilms in CF infection. Clin Rev Allergy Immunol 35:124–134. doi:10.1007/s12016-008-8079-9 18509765

[10] Borlee BR, Goldman AD, Murakami K, Samudrala R, Wozniak DJ, Parsek MR. 2010. Pseudomonas aeruginosa uses a cyclic-di-GMP-regulated adhesin to reinforce the biofilm extracellular matrix. Mol Microbiol 75:827–842. doi:10.1111/j.1365-2958.2009.06991.x 20088866 PMC2847200

[11] Rybtke M, Berthelsen J, Yang L, Høiby N, Givskov M, Tolker-Nielsen T. 2015. The LapG protein plays a role in Pseudomonas aeruginosa biofilm formation by controlling the presence of the CdrA adhesin on the cell surface. Microbiologyopen 4:917–930. doi:10.1002/mbo3.301 26458733 PMC4694147

[12] Colvin KM, Irie Y, Tart CS, Urbano R, Whitney JC, Ryder C, Howell PL, Wozniak DJ, Parsek MR. 2012. The Pel and Psl polysaccharides provide Pseudomonas aeruginosa structural redundancy within the biofilm matrix. Environ Microbiol 14:1913–1928. doi:10.1111/j.1462-2920.2011.02657.x 22176658 PMC3840794

[13] Baraquet C, Harwood CS. 2016. FleQ DNA binding consensus sequence revealed by studies of FleQ dependent regulation of biofilm gene expression in Pseudomonas aeruginosa. J Bacteriol 198:178–186. doi:10.1128/JB.00539-15 26483521 PMC4686206

[14] Baraquet C, Murakami K, Parsek MR, Harwood CS. 2012. The FleQ protein from Pseudomonas aeruginosa functions as both a repressor and an activator to control gene expression from the pel operon promoter in response to c-di-GMP. Nucleic Acids Res 40:7207–7218. doi:10.1093/nar/gks384 22581773 PMC3424551

[15] Heacock-Kang Y, Sun Z, Zarzycki-Siek J, McMillan IA, Norris MH, Bluhm AP, Cabanas D, Fogen D, Vo H, Donachie SP, Borlee BR, Sibley CD, Lewenza S, Schurr MJ, Schweizer HP, Hoang TT. 2017. Spatial transcriptomes within the Pseudomonas aeruginosa biofilm architecture. Mol Microbiol 106:976–985. doi:10.1111/mmi.13863 29030956 PMC5720903

[16] Williamson KS, Richards LA, Perez-Osorio AC, Pitts B, McInnerney K, Stewart PS, Franklin MJ. 2012. Heterogeneity in Pseudomonas aeruginosa biofilms includes expression of ribosome hibernation factors in the antibiotic-tolerant subpopulation and hypoxia-induced stress response in the metabolically active population. J Bacteriol 194:2062–2073. doi:10.1128/JB.00022-12 22343293 PMC3318454

[17] Liao J, Schurr MJ, Sauer K. 2013. The MerR-like regulator BrlR confers biofilm tolerance by activating multidrug efflux pumps in Pseudomonas aeruginosa biofilms. J Bacteriol 195:3352–3363. doi:10.1128/JB.00318-13 23687276 PMC3719540

[18] Mukherjee S, Moustafa D, Smith CD, Goldberg JB, Bassler BL. 2017. The RhlR quorum-sensing receptor controls Pseudomonas aeruginosa pathogenesis and biofilm development independently of its canonical homoserine lactone autoinducer. PLoS Pathog 13:e1006504. doi:10.1371/journal.ppat.1006504 28715477 PMC5531660

[19] Wilder CN, Diggle SP, Schuster M. 2011. Cooperation and cheating in Pseudomonas aeruginosa: the roles of the las, rhl and pqs quorum-sensing systems. ISME J 5:1332–1343. doi:10.1038/ismej.2011.13 21368905 PMC3146268

[20] Carey JN, Lamont S, Wozniak DJ, Dandekar AA, Parsek MR. 2024. Quorum sensing regulation of Psl polysaccharide production by Pseudomonas aeruginosa. J Bacteriol 206:e0031224. doi:10.1128/jb.00312-24 39530727 PMC11656772

[21] Persat A, Inclan YF, Engel JN, Stone HA, Gitai Z. 2015. Type IV pili mechanochemically regulate virulence factors in Pseudomonas aeruginosa. Proc Natl Acad Sci USA 112:7563–7568. doi:10.1073/pnas.1502025112 26041805 PMC4475988

[22] Park S, Sauer K. 2022. Controlling biofilm development through cyclic di-GMP signaling, p 69–94. In Pseudomonas aeruginosa: biology, pathogenesis and control strategies 10.1007/978-3-031-08491-1_3 PMC9891824 36258069

[23] Petrova OE, Sauer K. 2009. A novel signaling network essential for regulating Pseudomonas aeruginosa biofilm development. PLoS Pathog 5:e1000668. doi:10.1371/journal.ppat.1000668 19936057 PMC2774163

[24] Brencic A, McFarland KA, McManus HR, Castang S, Mogno I, Dove SL, Lory S. 2009. The GacS/GacA signal transduction system of Pseudomonas aeruginosa acts exclusively through its control over the transcription of the RsmY and RsmZ regulatory small RNAs. Mol Microbiol 73:434–445. doi:10.1111/j.1365-2958.2009.06782.x 19602144 PMC2761719

[25] Park S, Sauer K. 2021. SagS and its unorthodox contributions to Pseudomonas aeruginosa biofilm development. Biofilm 3:100059. doi:10.1016/j.bioflm.2021.100059 34729470 PMC8543379

[26] Mikkelsen H, Sivaneson M, Filloux A. 2011. Key two-component regulatory systems that control biofilm formation in Pseudomonas aeruginosa. Environ Microbiol 13:1666–1681. doi:10.1111/j.1462-2920.2011.02495.x 21554516

[27] Valentini M, Filloux A. 2016. Biofilms and cyclic di-GMP (c-di-GMP) signaling: lessons from Pseudomonas aeruginosa and other bacteria. J Biol Chem 291:12547–12555. doi:10.1074/jbc.R115.711507 27129226 PMC4933438

[28] Römling U, Galperin MY, Gomelsky M. 2013. Cyclic di-GMP: the first 25 years of a universal bacterial second messenger. Microbiol Mol Biol Rev 77:1–52. doi:10.1128/MMBR.00043-12 23471616 PMC3591986

[29] Jenal U, Reinders A, Lori C. 2017. Cyclic di-GMP: second messenger extraordinaire. Nat Rev Microbiol 15:271–284. doi:10.1038/nrmicro.2016.190 28163311

[30] Petrova OE, Gupta K, Liao J, Goodwine JS, Sauer K. 2017. Divide and conquer: the Pseudomonas aeruginosa two-component hybrid SagS enables biofilm formation and recalcitrance of biofilm cells to antimicrobial agents via distinct regulatory circuits. Environ Microbiol 19:2005–2024. doi:10.1111/1462-2920.13719 28263038 PMC5702475

[31] Petrova OE, Sauer K. 2011. SagS contributes to the motile-sessile switch and acts in concert with BfiSR to enable Pseudomonas aeruginosa biofilm formation. J Bacteriol 193:6614–6628. doi:10.1128/JB.00305-11 21949078 PMC3232883

[32] Hickman JW, Harwood CS. 2008. Identification of FleQ from Pseudomonas aeruginosa as a c-di-GMP-responsive transcription factor. Mol Microbiol 69:376–389. doi:10.1111/j.1365-2958.2008.06281.x 18485075 PMC2612001

[33] Gupta K, Marques CNH, Petrova OE, Sauer K. 2013. Antimicrobial tolerance of Pseudomonas aeruginosa biofilms is activated during an early developmental stage and requires the two-component hybrid SagS. J Bacteriol 195:4975–4987. doi:10.1128/JB.00732-13 23995639 PMC3807491

[34] Gupta K, Liao J, Petrova OE, Cherny KE, Sauer K. 2014. Elevated levels of the second messenger c-di-GMP contribute to antimicrobial resistance of Pseudomonas aeruginosa. Mol Microbiol 92:488–506. doi:10.1111/mmi.12587 24655293 PMC4029167

[35] Poudyal B, Sauer K. 2018. PA3177 encodes an active diguanylate cyclase that contributes to the biofilm antimicrobial tolerance but not biofilm formation by P. aeruginosa. Antimicrob Agents Chemother 62:e01049–01018. doi:10.1128/AAC.01049-18 30082282 PMC6153807

[36] Chambers JR, Liao J, Schurr MJ, Sauer K. 2014. BrlR from Pseudomonas aeruginosa is a c-di-GMP-responsive transcription factor. Mol Microbiol 92:471–487. doi:10.1111/mmi.12562 24612375 PMC4000578

[37] Oladosu VI, Park S, Sauer K. 2024. Flip the switch: the role of FleQ in modulating the transition between the free-living and sessile mode of growth in Pseudomonas aeruginosa. J Bacteriol 206:e0036523. doi:10.1128/jb.00365-23 38436566 PMC10955856

[38] Tseng BS, Zhang W, Harrison JJ, Quach TP, Song JL, Penterman J, Singh PK, Chopp DL, Packman AI, Parsek MR. 2013. The extracellular matrix protects Pseudomonas aeruginosa biofilms by limiting the penetration of tobramycin. Environ Microbiol 15:2865–2878. doi:10.1111/1462-2920.12155 23751003 PMC4045617

[39] Matsuyama BY, Krasteva PV, Baraquet C, Harwood CS, Sondermann H, Navarro M. 2016. Mechanistic insights into c-di-GMP–dependent control of the biofilm regulator FleQ from Pseudomonas aeruginosa. Proc Natl Acad Sci U S A 113:E209–E218. doi:10.1073/pnas.1523148113 26712005 PMC4720306

[40] Liao J, Sauer K. 2012. The MerR-like transcriptional regulator BrlR contributes to Pseudomonas aeruginosa biofilm tolerance. J Bacteriol 194:4823–4836. doi:10.1128/JB.00765-12 22730129 PMC3430307

[41] Poudyal B, Sauer K. 2018. The ABC of biofilm drug tolerance: the MerR-like regulator BrlR is an activator of ABC transport systems, with PA1874-77 contributing to the tolerance of Pseudomonas aeruginosa biofilms to tobramycin. Antimicrob Agents Chemother 62:e01981–01917. doi:10.1128/AAC.01981-17 29180529 PMC5786766

[42] Zhang L, Mah T-F. 2008. Involvement of a novel efflux system in biofilm-specific resistance to antibiotics. J Bacteriol 190:4447–4452. doi:10.1128/JB.01655-07 18469108 PMC2446775

[43] Petrova OE, Sauer K. 2010. The novel two-component regulatory system BfiSR regulates biofilm development by controlling the small RNA rsmZ through CafA. J Bacteriol 192:5275–5288. doi:10.1128/JB.00387-10 20656909 PMC2950493

[44] Hsu JL, Chen HC, Peng HL, Chang HY. 2008. Characterization of the histidine-containing phosphotransfer protein B-mediated multistep phosphorelay system in Pseudomonas aeruginosa PAO1. J Biol Chem 283:9933–9944. doi:10.1074/jbc.M708836200 18256026 PMC2442296

[45] Dingemans J, Poudyal B, Sondermann H, Sauer K. 2018. The yin and yang of SagS: distinct residues in the HmsP domain of SagS independently regulate biofilm formation and biofilm drug tolerance. mSphere 3:00192–00118. doi:10.1128/mSphere.00192-18 PMC5976881 29848761

[46] Chambers JR, Sauer K. 2013. The MerR-like regulator BrlR impairs Pseudomonas aeruginosa biofilm tolerance to colistin by repressing PhoPQ. J Bacteriol 195:4678–4688. doi:10.1128/JB.00834-13 23935054 PMC3807428

[47] Chambers JR, Cherny KE, Sauer K. 2017. Susceptibility of Pseudomonas aeruginosa dispersed cells to antimicrobial agents is dependent on the dispersion cue and class of the antimicrobial agent used. Antimicrob Agents Chemother 61:e00846–00817. doi:10.1128/AAC.00846-17 28971863 PMC5700346

[48] Ferreira T, Rasband W. 2011. ImageJ user guide. National Institutes of Health, USA.

[49] Winsor GL, Van Rossum T, Lo R, Khaira B, Whiteside MD, Hancock REW, Brinkman FSL. 2009. Pseudomonas genome database: facilitating user-friendly, comprehensive comparisons of microbial genomes. Nucleic Acids Res 37:D483–D488. doi:10.1093/nar/gkn861 18978025 PMC2686508

[50] Dingemans J, Al-Feghali RE, Sondermann H, Sauer K. 2019. Signal sensing and transduction are conserved between the periplasmic sensory domains of BifA and SagS. mSphere 4:e00442–00419. doi:10.1128/mSphere.00442-19 31366711 PMC6669338

[51] Miller J. 1972. Experiments in molecular genetics, p 352–355. Cold Spring Harbor Laboratory, Cold Spring Harbor, N.Y.

[52] Kulasekara HD, Ventre I, Kulasekara BR, Lazdunski A, Filloux A, Lory S. 2005. A novel two-component system controls the expression of Pseudomonas aeruginosa fimbrial cup genes. Mol Microbiol 55:368–380. doi:10.1111/j.1365-2958.2004.04402.x 15659157

[53] Karimova G, Ullmann A, Ladant D. 2000. A bacterial two-hybrid system that exploits A cAMP signaling cascade in Escherichia coli. Methods Enzymol 328:59–73. doi:10.1016/s0076-6879(00)28390-0 11075338

[54] Jacobs MA, Alwood A, Thaipisuttikul I, Spencer D, Haugen E, Ernst S, Will O, Kaul R, Raymond C, Levy R, Chun-Rong L, Guenthner D, Bovee D, Olson MV, Manoil C. 2003. Comprehensive transposon mutant library of Pseudomonas aeruginosa. Proc Natl Acad Sci USA 100:14339–14344. doi:10.1073/pnas.2036282100 14617778 PMC283593

[55] Newman JR, Fuqua C. 1999. Broad-host-range expression vectors that carry the l-arabinose-inducible Escherichia coli araBAD promoter and the araC regulator. Gene 227:197–203. doi:10.1016/S0378-1119(98)00601-5 10023058

[56] Kaneko Y, Thoendel M, Olakanmi O, Britigan BE, Singh PK. 2007. The transition metal gallium disrupts Pseudomonas aeruginosa iron metabolism and has antimicrobial and antibiofilm activity. J Clin Invest 117:877–888. doi:10.1172/JCI30783 17364024 PMC1810576

[57] Petrova OE, Cherny KE, Sauer K. 2014. The Pseudomonas aeruginosa diguanylate cyclase GcbA, a homolog of P. fluorescens GcbA, promotes initial attachment to surfaces, but not biofilm formation, via regulation of motility. J Bacteriol 196:2827–2841. doi:10.1128/JB.01628-14 24891445 PMC4135668

[58] Figurski DH, Helinski DR. 1979. Replication of an origin-containing derivative of plasmid RK2 dependent on a plasmid function provided in trans. Proc Natl Acad Sci USA 76:1648–1652. doi:10.1073/pnas.76.4.1648 377280 PMC383447

[59] Hoang TT, Karkhoff-Schweizer RR, Kutchma AJ, Schweizer HP. 1998. A broad-host-range Flp-FRT recombination system for site-specific excision of chromosomally-located DNA sequences: application for isolation of unmarked Pseudomonas aeruginosa mutants. Gene 212:77–86. doi:10.1016/s0378-1119(98)00130-9 9661666

[60] Schweizer HP, Hoang TT. 1995. An improved system for gene replacement and xylE fusion analysis in Pseudomonas aeruginosa. Gene 158:15–22. doi:10.1016/0378-1119(95)00055-b 7789804

[61] Sauer K, Camper AK. 2001. Characterization of phenotypic changes in Pseudomonas putida in response to surface-associated growth. J Bacteriol 183:6579–6589. doi:10.1128/JB.183.22.6579-6589.2001 11673428 PMC95489

[62] Sauer K, Camper AK, Ehrlich GD, Costerton JW, Davies DG. 2002. Pseudomonas aeruginosa displays multiple phenotypes during development as a biofilm. J Bacteriol 184:1140–1154. doi:10.1128/jb.184.4.1140-1154.2002 11807075 PMC134825

[63] Sauer K, Cullen MC, Rickard AH, Zeef LAH, Davies DG, Gilbert P. 2004. Characterization of nutrient-induced dispersion in Pseudomonas aeruginosa PAO1 biofilm. J Bacteriol 186:7312–7326. doi:10.1128/JB.186.21.7312-7326.2004 15489443 PMC523207

[64] Heydorn A, Nielsen AT, Hentzer M, Sternberg C, Givskov M, Ersbøll BK, Molin S. 2000. Quantification of biofilm structures by the novel computer program COMSTAT. Microbiology (Reading) 146 (Pt 10):2395–2407. doi:10.1099/00221287-146-10-2395 11021916

[65] Vorregaard M. 2008. Comstat2-a modern 3D image analysis environment for biofilms. Citeseer.

[66] Roy AB, Petrova OE, Sauer K. 2012. The phosphodiesterase DipA (PA5017) is essential for Pseudomonas aeruginosa biofilm dispersion. J Bacteriol 194:2904–2915. doi:10.1128/JB.05346-11 22493016 PMC3370607

[67] Basu Roy A, Petrova OE, Sauer K. 2013. Extraction and quantification of cyclic Di-GMP from Pseudomonas aeruginosa. Bio-protocol. Available from: http://www.bio-protocol.org/wenzhang.aspx?id=828 10.21769/bioprotoc.828 PMC4241849 25429368

[68] Battesti A, Bouveret E. 2012. The bacterial two-hybrid system based on adenylate cyclase reconstitution in Escherichia coli. Methods 58:325–334. doi:10.1016/j.ymeth.2012.07.018 22841567

[69] Karimova G, Pidoux J, Ullmann A, Ladant D. 1998. A bacterial two-hybrid system based on a reconstituted signal transduction pathway. Proc Natl Acad Sci USA 95:5752–5756. doi:10.1073/pnas.95.10.5752 9576956 PMC20451

[70] Livak KJ, Schmittgen TD. 2001. Analysis of relative gene expression data using real-time quantitative PCR and the 2^−ΔΔCT^ method. Methods 25:402–408. doi:10.1006/meth.2001.1262 11846609

